# Isolation and identification of *Enterococcus faecalis* membrane proteins using membrane shaving, 1D SDS/PAGE, and mass spectrometry

**DOI:** 10.1002/2211-5463.12075

**Published:** 2016-05-19

**Authors:** Peter Cathro, Peter McCarthy, Peter Hoffmann, Peter Zilm

**Affiliations:** ^1^ Oral Microbiology LaboratorySchool of DentistryThe University of AdelaideSouth AustraliaAustralia; ^2^ Neurovascular Research LaboratoryCentre for Cancer BiologyUniversity of South AustraliaAdelaideSouth AustraliaAustralia; ^3^ Adelaide Proteomics CentreThe University of AdelaideSouth AustraliaAustralia

**Keywords:** 1D SDS/PAGE, *Enterococcus faecalis*, mass spectrometry, membrane shaving, proteomics

## Abstract

*Enterococcus faecalis* is a significant nosocomial pathogen, which is able to survive in diverse environments and resist killing with antimicrobial therapies. The expression of cell membrane proteins play an important role in how bacteria respond to environmental stress. As such, the capacity to identify and study membrane protein expression is critical to our understanding of how specific proteins influence bacterial survival. Here, we describe a combined approach to identify membrane proteins of *E. faecalis* ATCC V583 using membranes fractionated by either 1D SDS/PAGE or membrane shaving, coupled with LC‐ESI mass spectrometry. We identified 222 membrane‐associated proteins, which represent approximately 24% of the predicted membrane‐associated proteome: 170 were isolated using 1D SDS/PAGE and 68 with membrane shaving, with 36 proteins being common to both the techniques. Of the proteins identified by membrane shaving, 97% were membrane‐associated with the majority being integral membrane proteins (89%). Most of the proteins identified with known physiology are involved with transportation across the membrane. The combined 1D SDS/PAGE and membrane shaving approach has produced the greatest number of membrane proteins identified from *E. faecalis* to date. These protocols will aid future researchers investigating changes in the membrane proteome of *E. faecalis* by improving our understanding of how *E. faecalis* adapts and responds to its environment.

**Abbreviations**
1D SDS/PAGE: one‐dimensional sodium dodecyl sulfate–polyacrylamide gel electrophoresis
ACN: acetonitrile
DTT: dithiothreitol
GRAVY: grand average of hydropathy
HCl: hydrochloric acid
IAA: iodoacetamide
IMPs: integral membrane proteins
LC‐ESI: liquid chromatography‐electrospray ionization
PBS: phosphate‐buffered saline
TBP: tributylphosphine
TEAB: triethylammonium bicarbonate
TMD: transmembrane domain

The cytoplasmic membrane of a bacterium plays a crucial role in homeostasis and the ability to invade, adapt, and respond to the extracellular environment. Membrane proteins that are expressed have a wide variety of functions including, nutrient uptake, response to environmental stress, adhesion, virulence, biofilm formation, and antibiotic resistance 1, 2. Integral membrane proteins are also important in the initiation of signal transduction pathways, allowing the bacterial cell to adjust its physiology to changes in the external environment 3.

*Enterococcus faecalis* is a Gram‐positive facultative anaerobe that is used within the food industry in certain cheeses and sausages and is a commensal organism within the gastrointestinal tract 4, 5. However, it has also been recovered from patients suffering endocarditis, bacteremia, urinary tract infections, wound infections, meningitis 6, and is often present in teeth with root fillings that have evidence of persistent infection 7. *Enterococcus faecalis* demonstrates a remarkable ability to survive a wide range of environmental conditions including the extremes of gastric acid and high pH used in dental medicaments 8. The complete genome sequence has been published by Paulsen *et al*. 1 but only a fraction of the 781 (approximately) membrane proteins have been isolated and identified (http://www.cmbi.ru.nl/locatep-db/cgi-bin/locatepdb.py). Nine membrane‐embedded proteins were identified in *E. faecalis* V583 by Bøhle *et al*. 9 and 64 in *E. faecalis* OG1X by Maddalo *et al*. 10; the latter being the most comprehensive study of the membrane proteome to date. The diversity of function makes membrane proteins potential targets for the development of drugs or medicaments, which may improve the efficacy of current therapeutic strategies.

Proteomic studies of cell membrane proteins are hampered by their low abundance and the hydrophobic nature of the transmembrane domain 11. Standard proteomic approaches combining 1D or 2D polyacrylamide gel electrophoresis (PAGE) and mass spectrometry generally use strong chaotropic agents or strong detergents (traditionally SDS) to solubilize membrane proteins, which are ultimately poorly represented against highly abundant cytoplasmic proteins. A number of fractionation protocols have therefore been used to enrich for bacterial membrane proteins before identification by mass spectrometry 3.

Enrichment of membrane proteins is an obvious approach to significantly reduce sample complexity and improve the resolution of the bacterial membrane proteome. This is typically achieved by isolation of membranes following cell lysis and by differential centrifugation or precipitation with cold sodium carbonate. Sodium carbonate linearizes and precipitates membranes which then allows solubilization of peripheral and integral membrane proteins in detergents 3 which can then be separated using techniques, such as anion exchange chromatography 10, and 1D SDS/PAGE 12. While membrane enrichment reduces sample complexity, the associated downstream separation steps can still produce losses of poorly solubilized and/or highly hydrophobic membrane proteins. Proteins containing multiple transmembrane domains are particularly difficult to recover and are rarely identified, if at all 12. Accordingly, methods that reduce sample complexity without introducing sample‐hungry fractionation steps are highly desirable. Recently, the generation and isolation of transmembrane domain (TMD) peptides using membrane shaving have been shown to complement other membrane enrichment techniques 11, 12. Briefly, membrane shaving involves treating extracted membranes with proteinase K to digest exposed hydrophilic domains leaving behind only the membrane‐embedded domains which are then digested using chymotrypsin. The transmembrane domain (TMD) peptides are then separated and identified directly using mass spectrometry 11.

Recently, Wolff *et al*. 12 compared 1D SDS/PAGE followed by LC‐MS/MS, strong cation exchange (SCX) chromatography followed by LC‐MS/MS, and membrane shaving followed by LC‐MS/MS analysis on *Staphylococcus aureus*. They identified 271 integral membrane proteins (IMPs) and found 1D SDS/PAGE and membrane shaving approaches to be highly complementary. Membrane shaving yielded almost exclusively IMPs (96.7%).

In this present study, we have adapted and combined the protocols used by Wolff *et al*. 12 with the aim of increasing the current resolution and identification of the membrane proteome of *E. faecalis*.

## Materials and methods

### Growth conditions

*Enterococcus faecalis* ATCC V583 strain was purchased from Cryosite (NSW, Australia) and maintained on Columbia blood agar (Oxoid, Melbourne, Australia) at 37 °C. Culture purity was periodically checked by culturing onto bile aesculin agar (Oxoid). About 1000 mL of sterile Todd Hewitt broth (THB) (Oxoid), was inoculated with 1 mL of an overnight broth and incubated at 37 °C for 3 days. Bacteria were harvested by centrifugation (6000 ***g***), at 4 °C for 20 min. Cells were washed twice with saline (0.9% w/v) at 4 °C and cells were finally resuspended in 12 mL of ice cold saline. Cells were lysed by two passes (60 000 kPa) through a SLM Aminco French Press (Thermo Fisher, Waltham, MA, USA). Endogenous proteinase activity was controlled during lysis by the addition of 100 μL of bacterial protease inhibitor cocktail (Sigma, St. Louis, MO, USA). Nucleic acids were then degraded by the addition of deoxyribonuclease I (2000 Units), ribonuclease A (1000 Units), and MgCl_2_ (50 mm), and incubated on ice for 60 min. Intact cells were removed by centrifuging twice (8000 ***g*** at 4 °C for 5 min) and removing the supernatant.

### Membrane isolation and 1D SDS/PAGE

The protein concentration of the cell‐free lysate was determined using the Coomassie Plus (Bradford) Assay Kit (Thermo Fisher) and membrane proteins were purified from 100 mg of crude protein. Following ultracentrifugation (100 000 ***g***, 60 min, 4 °C), the pellet was homogenized in 8 mL high salt buffer (20 mM Tris/HCl, pH 7.5, 10mM EDTA, 1M NaCl) containing protease inhibitor and incubated for 30 min at 4 °C on a rotary shaker. The solution was then ultracentrifuged (100 000 ***g***, 60 min, 4 °C) and the pellet was homogenized in 8 mL 100 mm Na_2_CO_3_‐HCl, pH11, 10 mm EDTA, 100 mm NaCl. Following ultracentrifugation, (100 000 ***g***, 60 min, 4 °C) the pellet containing the bacterial membrane was washed with 8 mL 50 mm triethylammonium bicarbonate (TEAB) pH7.8 buffer and then ultracentrifuged (100 000 ***g***, 60 min, 4 °C) before the pellet was homogenized in 500 μL 50 mm TEAB, pH7.8 buffer. The protein concentration was determined according to the Bradford assay described above. An aliquot containing 20 μg of the purified membrane protein was reduced with 4 mm tributylphosphine (TBP) (BioRad, Hercules, CA, USA) at 50 °C for 30 min. Alkylation of the samples was performed with 10 mm iodoacetamide (BioRad) in the dark for 30 min.

The sample (500 μL) was then purified using a 2D clean‐up kit (BioRad) following the manufacturer's instructions.

Twenty microlitre was loaded onto a Criterion TGX Precast gel (Biorad) and separation performed at 200V constant voltage. After completion, the gel lane was cut into 12 equal‐sized pieces and subjected to in‐gel tryptic digestion. Briefly, the bands were reduced with 10 mm dithiothreitol (DTT) in 100 mm ammonium bicarbonate. Alkylation of proteins was performed using 55 mm iodoacetamide (IAA) in 100 mm ammonium bicarbonate. Overnight digestion was performed using 100 ng of sequencing grade modified trypsin (Promega Corporation, Madison, WI, USA) in 5 mm ammonium bicarbonate containing 10% acetonitrile (ACN). The digest was stopped by addition of 30 μL of 1% formic acid. Two further extractions using 50 μL of 1% formic acid in 50% ACN and 50 μL of 100% ACN with sonication for 15 min were performed. For each gel piece, extracts were pooled. The volumes of the resulting peptide extracts were reduced by vacuum centrifugation to approximately 2 μL and then resuspended in 0.1% trifluoroacetic acid (TFA) in 2% ACN to a total volume of ~ 10 μL.

### Membrane shaving

The protein concentration of the cell‐free lysate was determined as described previously and adjusted to 1 mg·mL^−1^ with saline. An aliquot containing 60 mg of protein was pelleted by ultracentrifugation (100 000 ***g*** at 4 °C for 1 h) and the membranes were washed in phosphate‐buffered saline (PBS) followed by further ultracentrifugation (100 000 ***g*** at 4 °C for 1 h). The pellet was carefully resuspended in 1000 μL of carbonate buffer (200 mm Na_2_CO_3_ pH11.0) using an insulin syringe to homogenize the pellet. The sample was incubated on ice for 1 h and homogenized every 15 min. The protein concentration of the homogenized pellet was determined and the concentration was adjusted to 1 mg·mL^−1^ with carbonate buffer. With the sample at room temperature, solid urea (BioRad) was added to a concentration of 8M. Samples were reduced with 4 mm tributylphosphine (TBP) (BioRad) at 50 °C for 30 min. Alkylation of the samples was performed with 10 mm IAA in the dark for 30 min. Proteinase K (Sigma) was then added to the sample in an enzyme/protein ratio of 1 : 50 and incubated overnight at 35 °C on a shaker. An equal volume of 10% ACN (Ajax Finechem‐Thermo Fisher Scientific, Sydney, Australia) in water was added and the sample was cooled on ice for 15 min. Samples were then ultracentrifuged (100 000 ***g*** at 4 °C for 1 h), and the supernatant was discarded and the pellet was rinsed with 50 mm TEAB (pH 8.4–8.6) to remove residual urea. Membranes were then pelleted by centrifugation (100 000 ***g***) at 4 °C for 1 h.

The pellet was resuspended in 200 μL of TEAB 10 mm calcium chloride and 0.5% RapiGest (Waters, Milford, MA, USA). About, 4 μg of chymotrypsin (Sigma) was added and digestion was performed for 6 h at 30 °C (with shaking). RapiGest was removed by incubation in 0.25M HCl solution (pH < 2) for 45 min at 37 °C.

The sample was then centrifuged three times (20 000 ***g*** at 4 °C for 15 min) each time collecting the supernatant‐containing peptides.

The resultant supernatant was then analyzed with LC‐MS/MS. Peptides were desalted and concentrated using C18 spin column (Pierce, Rockford, IL, USA). Peptides were eluted using ACN/TFA/H2O (70 : 0.5 : 29.5, v/v) and freeze dried. The lyophilized peptides were resuspended using ACN/TFA/H2O (2 : 0.1 : 97.9, v/v). The volumes of the resulting peptide extracts were reduced by vacuum centrifugation to approximately 2 μL then resuspended with 0.1% TFA in 2% ACN to a total volume of ~ 10 μL.

### Liquid chromatography–electrospray ionization tandem mass spectrometry

Peptides were separated on an Ultimate 3000 HPLC system (Dionex, Sunnyvale, CA, USA) coupled to a LTQ Orbitrap XL mass spectrometer (Thermo Fisher Scientific, Bremen, Germany). Samples (5 μL) were injected into a trapping column (Acclaim PepMap100, C18, pore size 100 Å, particle size 3 μm, 75 μm ID × 2 cm length) and then resolved on a separation column (Acclaim PepMap RSLC, C18, pore size 100 Å, particle size 2 μm, 75 μm inner diameter (ID) × 15 cm length). The HPLC solvent A was 2% ACN, 0.1% FA in water and solvent B was 80% ACN, 0.1% FA in water. Peptides were eluted at 300 nL·min^−1^ flow rate with the following 100 min gradient: 4% B for 10 min, gradient to 40% B over 50 min, gradient to 90% B in 20 min, 90% B for 10 min, gradient from 90% to 4% B in 30 s, 4% B for 19.5 min. The LTQ Orbitrap XL instrument was operated in data‐dependent mode to automatically switch between full scan MS and MS/MS acquisition. Instrument control was through thermo tune plus and xcalibur software (Thermo Scientific, Bremen, Germany).

A full scan MS spectra (*m*/*z* = 300–1700) were acquired in the Orbitrap analyzer and resolution in the Orbitrap system was set to *r* = 60 000. The standard mass spectrometric conditions for all experiments were: spray voltage, 1.25 kV; no sheath and auxiliary gas flow; heated capillary temperature, 200 °C; predictive automatic gain control (AGC) enabled; and an S‐lens RF level of 50–60%. All unassigned charge states and charge state of + 1 were rejected. The six most intense peptide ions with charge states ≥ 2 and minimum signal intensity of 1000 were sequentially isolated and fragmented in the high‐pressure linear ion trap by low‐energy CID. An activation *q* = 0.25, activation time of 30 ms, and normalized collision energy of 35% were used. The resulting fragment ions were scanned out in the low‐pressure ion trap at the ‘normal scan rate’ (33 333 amu·s^−1^) and recorded with the secondary electron multipliers.

Raw data files were subjected to the proteome discoverer software (Thermo Scientific) to set up the workflow, and files were then submitted to mascot 13 (Perkins *et al*.) (Version 2.2; Matrix Science Inc., Boston, USA, 2007) by the Proteome Discoverer Daemon (Thermo Scientific). Peak lists in the range from 350 *m*/*z* to 5000 *m*/*z* were searched against the NCBInr database. The taxonomy filter search for Mammalia and *E. faecalis* were used with the enzyme setting of trypsin for the 1D gel pieces, and the taxonomy filter search for bacteria and enzyme setting of chymotrypsin for the membrane shaving samples. For peptide identifications, we set a minimum expectation value of *P* < 0.05. We used a machine learning algorithm called Percolator (Brosch *et al*.) 14 within mascot in all searches and all resulting peptide IDs fell within an FDR of < 2%. Protein identifications were made on the basis of having at least two unique peptides that satisfied the above criteria. These unique peptides were required to have different sequences or different variations of the same sequence, for example, containing a modified residue or missed cleavage site. Multiple charge states were not considered as unique.

### Protein analysis

The proteins identified from both 1D SDS/PAGE and membrane shaving isolation techniques were searched using the Locate P database (http://www.cmbi.ru.nl/locatep-db/cgi-bin/locatepdb.py) to determine the predicted localization. The total number of membrane‐associated proteins and intracellular proteins were determined for each membrane enrichment protocol and for proteins common to both techniques. The membrane‐associated proteins were then cross‐matched with the published results of Paulsen *et al*. 1, Reffuveille *et al*. 15, Maddalo *et al*. 10, and Bøhle *et al*. 9 for comparison with previous identifications and predicted roles in biofilm formation, stress, and virulence.

The NCBI protein database (http://www.ncbi.nlm.nih.gov) was searched using the gene name derived from Locate P to obtain the FASTA format for each membrane‐associated protein, which was then used to search with tmhmm server v.2.0 (http://www.cbs.dtu.dk/services/TMHMM/) for a prediction of the number of transmembrane helices and also searched with expasy protparam (http://web.expasy.org/protparam/) for the GRAVY scores.

## Results and Discussion

A total of 513 proteins were identified with both 1D SDS/PAGE and membrane shaving protocols. The predicted localization of the proteins identified was categorized with both Locate P and the Locate P prediction by SwissProt classification (Table 1). The IMPs include Locate P predictions of: Multi‐transmembrane; Multi‐transmembrane (lipid modified N‐termini), and N‐terminally membrane anchored locations. For the purposes of this study, the membrane‐associated proteins include the lipid‐anchored locations in addition to the IMPs.

**Table 1:** Predicted localization and number of identified membrane proteins using 1D SDS/PAGE and membrane shaving

Predicted localization	Locate P prediction by SwissProt Classification	1D SDS/PAGE	Shaving	1D and Shaving	No. of identified proteins	No. in the *E. faecalis* V583 genome^a^	Percent of predicted identified
Multi‐transmembrane	Membrane	92	58	32	118	581	20
Multi‐transmembrane (lipid modified N‐termini)	Membrane	3	1	1	3	7	43
N‐terminally membrane anchored	Membrane	41	3	1	43	193	22
Lipid anchor	Extracellular	34	6	2	38	74	51
LPxTG cell wall anchor	Cell wall	1	1		2	42	5
Secreted	Extracellular	9	0	0	9	55	16
Intracellular	Cytoplasmic	299	1		300	2303	13

a Data from LocateP 16.

Four hundred and seventy‐nine proteins were identified using 1D SDS/PAGE with 170 of these predicted to be membrane‐associated (35.5%) and 299 intracellular (62.4%). The membrane shaving protocol yielded a total of 70 proteins with 68 (97%) predicted to be membrane‐associated, one (1.4%) intracellular, and one (1.4%) attached to the cell wall. There were 36 membrane‐associated proteins that were common to both 1D SDS/PAGE and membrane shaving approaches giving a total of 202 unique membrane‐associated proteins. This represents 24% of the total 855 predicted proteins 16 (Table S1). Of the 202 membrane‐associated proteins recovered, 164 were IMPs which represent 21% of the 781 predicted IMPs. In addition to membrane‐associated proteins, two proteins were predicted to be located on the cell wall, one from each protocol, and there were nine secreted proteins identified using 1D SDS/PAGE. In total, 213 proteins that were not cytosolic were identified.

Our 1D SDS/PAGE and membrane shaving protocols resulted in 58 and 25 proteins, respectively that were common to the membrane‐associated proteins identified by Maddalo *et al*. 10 and Bøhle *et al*. 9 with 15 proteins common to both protocols. A total of 145 proteins therefore were unique to the present study (Table S1).

Ballering *et al*. 17 described 68 genetic loci predicted to be involved in biofilm formation by *E. faecalis*. Our 1D SDS/PAGE and membrane shaving protocols identified the expression of four and five corresponding proteins, respectively, with two being common to both protocols (Table S1).

Paulsen *et al*. 1 genomic study predicted 50 membrane proteins played a role in the organism's stress response. In the present study, 12 and 6 proteins were identified using 1D SDS/PAGE and membrane shaving, respectively, with five identified in both protocols (Table S1).

Of the 148 proteins in *E. faecalis* implicated in virulence, 1, 15 28 and 7 were identified using 1D SDS/PAGE and membrane shaving, with two being common to both protocols. The physiological classification of identified membrane‐associated proteins was determined by cross‐referencing with Maddalo *et al*. 10, Paulsen *et al*. 1, 15, and Wolff *et al*. 12. Of the 213 proteins with known function, the majority are involved with membrane transport (Table 2).

**Table 2:** Physiological classification of the 213 membrane proteins identified from 1D SDS/PAGE and membrane shaving protocols

Function	1D SDS/PAGE	Membrane Shaving	Common to both	Total	Percentage of all membrane‐associated proteins
Transport and binding	40	33	16	57	26.76
Virulence	28	7	2	33	15.49
Protein translocation and processing	7	4	1	10	4.69
Stress	10	4	4	10	4.69
Metabolism	7			7	3.29
Miscellaneous	10	0		10	4.69
Cell membrane/cell wall division	9	2	1	10	4.69
Unknown	57	16	7	76	35.68

**Figure 1:**
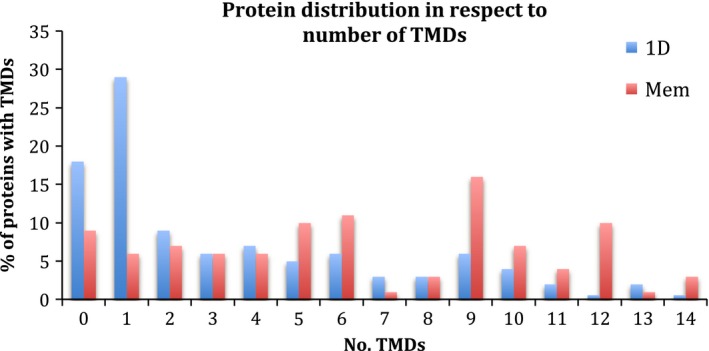
Allocation of membrane‐associated proteins in respect to their number of TMDs.

The 1D SDS/PAGE protocol favored the recovery of proteins with a smaller number of TMDs, whereas the membrane shaving protocol was useful in recovering proteins within the full range of 0–14 TMDs, but especially those with a higher number. The percentage of proteins identified with the various number of TMDs is reported according to the isolation protocol (Fig. 1).

**Figure 2:**
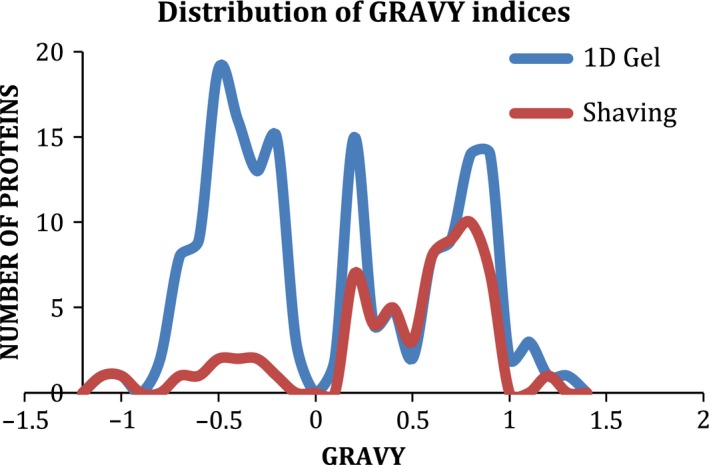
Frequency of GRAVY indices of membrane‐associated proteins recovered by 1D SDS/PAGE and membrane shaving protocols.

The GRAVY scores (the sum of hydropathy values of all amino acids divided by the protein length) given for proteins identified by 1D SDS/PAGE and membrane shaving are shown in Fig. 2.

In the present study, the combined approaches of Na_2_CO_3_/1D SDS/PAGE and membrane shaving have identified approximately 24% of the theoretical membrane proteome of *E. faecalis* V583. To our knowledge, this is the best recovery to date 16 and is approximately twice that of previous reports 10. A total of 145 proteins identified in this study have not been previously published 9, 10, 18.

Maintaining intact cells as spheroplasts, or cell lysis prior to membrane enrichment are the two main approaches used to identify surface attached, secreted, or cell membrane proteins. Bøhle *et al*. 9 employed proteolytic shaving of the intact bacterial cells with trypsin and recovered 36 surface‐located proteins, of those with surface‐located/exposed domains, three (0.5%) were annotated as integral membrane proteins 9. The low recovery was thought to be due to limited accessibility to the proteins and the limited ability of trypsin to penetrate the cell wall 9. Alternatively, the ability of trypsin to cleave sites in membrane proteins necessary for mass spectrometry preparation could also limit detection 12. In contrast to the intact cell methods, Maddalo *et al*. 10 lysed the cells with a French Press before membrane purification and enriched cell membranes by ultracentrifugation. In a similar fashion, we used this method to create the crude membrane extract in our study and precipitated the cell membrane using carbonate buffer as previously described 11, 12, 19. Enrichment with sodium carbonate has been shown to linearize and precipitate membranes and allows solubilization of peripheral and transmembrane proteins in strong detergents 3. It has been effective for Gram‐negative organisms but as a stand‐alone technique after cell lysis, it is less effective for Gram‐positive organisms due to their thick cell wall 3.

Membrane‐embedded proteins are especially difficult to recover due to the hydrophobic nature of the transmembrane domain. Following purification of the cell membrane, Maddalo *et al*. 10 separated the proteins using anion exchange chromatography and identified them by mass spectrometry. One hundred and two proteins were resolved with 64 (63%) identified as membrane‐embedded. The authors predicted that they had experimentally identified ~ 10% of the membrane‐embedded proteome of strain OG1X, which was the largest recovery of such proteins at the time. The 102 proteins identified could be classified as: 64 membrane‐embedded (63%); 9 lipoproteins (9%); 16 soluble components of membrane proteins complexes (16%); and 13 were soluble with no predicted membrane association (13%).

From *in silico* analysis, there are 781 predicted membrane‐embedded proteins in the *E. faecalis* V583 genome (http://www.cmbi.ru.nl/locatep-db/cgi-bin/locatepdb.py) and in the present study, the combined approaches resolved 21% (164 proteins).

The 1D SDS/PAGE approach resulted in a total number of 479 proteins identified with 170 being membrane‐associated (35.5%) and 299 intracellular. This is a very similar result to Wolff *et al*. 12 for *S. aureus* who reported 572 proteins with 179 being IMPs (31.3%).

In the present study, we have combined two complementary membrane fractionation techniques to isolate and identify the highest number of membrane‐associated proteins from *E. faecalis*. Wolff *et al*. 12 identified 182 proteins in *S. aureus* using membrane shaving, of which 176 (96.7%) were determined to be IMPs. Our recovery of IMPs using this protocol was much lower with only 68 proteins recovered; however, the proportion of proteins being IMPs was similar (97%). The discrepancy in the total number of proteins identified could be due to the mass spectrometry search parameters used, for example, setting the number of missed cleavages. If the digest is not completely perfect and peptides remain with intact cleavage sites, increasing the level of missed cleavages increases the number of calculated peptide masses to be matched against the experimental data. However, this increases the number of random matches and so reduces discrimination (http://www.matrixscience.com/help/search_field_help.html). Wolff *et al*. 12 searched with no enzyme specificity and with chymotrypsin allowing four missed cleavage sites. In contrast to improve the reliability of identification, we elected to search allowing two missed cleavages and only with chymotrypsin.

Highlighting the complementary nature of the isolation protocols, 1D SDS/PAGE favored the recovery of proteins with a lower number of TMDs and negative GRAVY scores, indicative of hydrophilic proteins. The protocol was superior for isolating N‐terminally membrane anchored (with usually one TMD) and lipid‐anchored proteins. Membrane shaving was especially good at the recovery of proteins with a large number of TMDs and identified predominantly hydrophobic proteins with a positive GRAVY score which demonstrates that this approach is particularly suitable for the identification of very hydrophobic proteins and is consistent with the analysis on *S. aureus* 12. In the present study, 1D SDS/PAGE produced the largest recovery of proteins in all cell locations including the intracellular region. In contrast, membrane shaving recovered only one intracellular protein with the majority predicted to be multi‐transmembrane or N‐terminally membrane anchored (~ 89%). Proteinase K was used on purified membrane extracts (ultracentrifugation and carbonate precipitation) thus targeting protein domains that are surface exposed 3. An acid‐labile detergent (Rapigest) was then used to dissolve the hydrophobic bilayer of the membrane and chymotrypsin added to further digest the liberated membrane‐spanning peptides, which were then analyzed using LC‐ESI mass spectrometry.

The majority of the identified integral membrane proteins are described as being involved in transport and binding proteins (28.2%). The high incidence of proteins dedicated to transport and a large number of proteins with unknown function is a similar finding to Maddalo *et al*. 10 and this is consistent with the large theoretical number of predicted transport membrane proteins in the proteome 1.

The total number of proteins expressed or recovered may vary according to the growth conditions or protein extraction protocols and likely contributes to some of the differences between the present and published studies. Sixty‐seven proteins identified in the present study were common to Maddalo *et al*. 10 and Bøhle *et al*. 9. Ballering *et al*. 17 carried out a comprehensive analysis of the genetic determinants of biofilm formation in the core genome of *E. faecalis*. Of the 68 genes identified by Ballering *et al*. 17, Maddalo *et al*. 10 identified six of these membrane proteins, while this study identified nine.

Paulsen *et al*. 1 reported the complete genome sequence of *E. faecalis* V583 and predicted 49 genes from the whole genome to have a potential role in the organism's stress response. This study identified one membrane protein associated with oxidative stress [EF3257]; eight for osmotic stress [EF0295, EF0568, EF0875, EF1493, EF1494, EF2612, EF2613, EF2614]; and three for metal‐ion resistance [EF1519, EF1938, EF2623]. This represents 24% of the predicted stress proteins. In addition to the virulence proteins determined by Paulsen *et al*. 1, Reffuveille *et al*. 15 reviewed the identification of lipoprotein‐encoding genes and their potential involvement in virulence. Of the virulence‐related genes predicted to be surface exposed, this study identified thirty‐three genes. Growth conditions in the present study could be considered ideal in terms of nutrient availability and temperature so it is perhaps not surprising that the recovery of proteins associated with roles in stress or virulence was low.

A fundamental consideration in identifying membrane proteins is to limit the contamination by highly abundant cytosolic proteins. The formation of spheroplasts was thought to reduce cytosolic protein contamination. Seven of the 27 proteins recovered by Benachour *et al*. 20 and 34 of the 69 recovered by Bøhle *et al*. 9 were identified as cytosolic proteins. This may reflect the intracellular association of these proteins with the cell membrane, or alternatively, may have been due to cell lysis prior to treatment with trypsin. The released cytosolic proteins may then have reassociated with the cell envelope and escaped proteolytic degradation. In this study, the 1D SDS/PAGE protocol resulted in 299 intracellular (cytosolic) proteins identified despite membrane precipitation. In contrast, membrane shaving appeared to be an excellent method to reduce cytoplasmic contamination as only one protein (EF021) was identified. EF021 is a 50S ribosomal protein, and is one of the 33 cytosolic proteins identified by Bøhle *et al*. 9

## Conclusion

A workflow combining 1D SDS/PAGE and membrane shaving was successful in the recovery of integral membrane proteins from *E. faecalis* V583. Of the 202 membrane‐associated proteins identified, 81% were membrane‐embedded and represents approximately 21% of the predicted membrane‐embedded proteome. These protocols will form a basis for further research into *E. faecalis* by investigating proteins expression under different growth conditions and aid our understanding how *E. faecalis* adapts to its environment.

## Author contributions

PC conducted all hands‐on experimental work and drafted the manuscript. The work is a component of his PhD undertaken at the University of Adelaide. PZ participated in the design and provided advice on the microbiological and proteomic aspects; and participation in the write‐up and submission process. PMcC conducted hands‐on proteomic experimental work and participation in the write‐up and submission process. PMcC and PH participated in the design of the experiments and provided advice on the proteomic component. All authors read and approved the final manuscript.

## Supporting information


**Table S1.** Membrane‐associated proteins. Protein in 1D gel bands common to membrane shaving protocol are highlighted in bold; (PI) proteins identified by (M) Maddalo *et al*. 10 and (B) Bøhle *et al*. 9; (Bio) Proteins reported to be involved in biofilm formation (Ballering *et al*. 17); (S) stress as reported by (P) Paulsen *et al*. 1; (V) virulence as reported by (P) Paulsen *et al*. 1 & (R) Reffuveille *et al*. 15).
